# Face‐Looking as a Real‐Time Process in Mind‐Mindedness: Timely Coordination Between Mothers' Gaze on Infants' Faces and Mind‐Related Comments

**DOI:** 10.1111/infa.12644

**Published:** 2024-12-01

**Authors:** Hiroki Yamamoto, Nagomi Sunahara, Yasuhiro Kanakogi

**Affiliations:** ^1^ Graduate School of Human Sciences Osaka University Suita Osaka Japan; ^2^ Department of Psychological and Brain Sciences Indiana University Bloomington Indiana USA; ^3^ Japan Society for the Promotion of Science Chiyoda‐ku Tokyo Japan

**Keywords:** face‐looking, infant‐caregiver interaction, maternal gaze, mentalization, mind‐mindedness

## Abstract

Maternal mind‐mindedness refers to a caregiver's tendency to respond to their infants as individuals with their own thoughts, feelings, desires, and beliefs. Although previous studies have focused on maternal speech in quantifying mind‐mindedness, maternal mind‐mindedness should manifest not only as mind‐related comments but also through non‐verbal behaviors during infant‐mother interactions. In this study, we investigated the relationship between maternal gaze at the infant's face and typical verbal measurement of mind‐mindedness in free‐flowing interactions. Forty 11‐ to 13‐month‐old infants and their mothers participated in the study; the mothers were asked to wear a head‐mounted eye tracker to measure their gaze during infant‐mother free‐play interactions. We measured the proportion of time mothers looked at the infant's face when it was present in the mother's field of view and examined the relationship between the face‐looking proportion and verbal measurement of mothers' mind‐mindedness. Mothers who displayed appropriate mind‐related comments looked at the faces of their infants more frequently. Moreover, their looking was coordinated in a timely manner with appropriate mind‐related comments compared with other comments. Our findings suggest that mothers looking at infants' faces supports comments regarding infants' mental states and shed new light on real‐time behaviors underlying mothers' mentalization processes.

## 1 Introduction

Mind‐mindedness pertains to a caregiver's tendency to represent and respond to their infants as individuals with their own thoughts, feelings, desires, and beliefs during infant‐caregiver interactions (Meins et al. 2001). Mind‐mindedness is typically operationalized through the appropriateness of caregivers' comments regarding their infants' putative internal states during infant‐caregiver interactions (Meins et al. 2001, 2012). Research over the last 2 decades has shown links between caregivers' ability to be mind‐minded about their children and children's development. Particularly, positive associations have been found between mind‐mindedness and secure attachment (Meins et al. 2001, 2012; Miller et al. 2019). In addition, maternal mind‐mindedness in early infancy can predict children's cognitive and social development (Aldrich, Chen, and Alfieri 2021). Individual differences in maternal mind‐mindedness are associated with differences in the theory of mind (Meins et al. 2002, 2003), understanding of emotions (Centifanti, Meins, and Fernyhough 2016), social competence (Colonnesi et al. 2019), language development (Bernier, Calkins, and Bell 2016; Meins et al. 2013), and educational attainment (Meins, Fernyhough, and Centifanti 2019).

Maternal mind‐mindedness is stable over the first 2 years of infancy (Meins et al. 2003, 2011) and unrelated to infant temperament (Meins et al. 2011), socioeconomic status (Meins et al. 1998), and maternal education (Meins et al. 2001, 2003). Furthermore, maternal mind‐mindedness is correlated with other types of caregiver behavior such as maternal mirroring of infant behavior (Bigelow et al. 2015) and parent‐embodied mentalizing (PEM), which is the caregiver's non‐verbal appreciation of the infant's mind reflected in their bodily movements (Shai and Meins 2018). In addition, previous research on mind‐mindedness (Miens 2013; Zeegers et al. 2017) has found robust associations between mind‐mindedness and maternal sensitivity, defined as the awareness and correct interpretation of the infant's cues and a contingent and appropriate response to these signals (Ainsworth, Bell, and Stayton 1974). Thus, maternal mind‐mindedness includes not only the caregiver's mentalization ability to infer their infant's mental state but also the caregiver's sensitivity to their infants.

When quantifying mind‐mindedness as caregivers' sensitivity to their infants, most previous studies focused on maternal speech (Meins et al. 2002, 2012). However, as mind‐mindedness was originally defined as sensitivity to infants' internal states, the mother's sensitivity should manifest in non‐verbal behaviors, as well as mind‐related comments in real‐time infant‐mother interactions. Although previous studies have reported some non‐verbal behaviors associated with mind‐related comments (Bigelow et al. 2015; Shai and Meins 2018), the associations were assessed as between‐individual variations rather than within‐individual cooccurrences. Therefore, little is known about what type of non‐verbal behavior displayed by mothers is coordinated in a timely manner with mind‐related comments in real‐time infant‐mother interactions. That is, how do mothers behave non‐verbally to appropriately infer and respond to their infants' mental states in real‐time interactions during which the infant's interests fluctuate from moment to moment? To understand mind‐mindedness as a real‐time process of inferring infants' current mental states, it is important to elucidate how mothers' non‐verbal behaviors are associated with their mind‐related comments in social interactions.

As a non‐verbal behavior related to the real‐time maternal mentalization process, we focused on looking at others' faces. Gazing at others is commonly referred to as social attention and has been studied not only in adults but also in a wide range of participants, including infants (e.g., Frank, Vul, and Johnson 2009), individuals with autism (e.g., Klin et al. 2002), and great apes (e.g., Kano, Call, and Tomonaga 2012). In social interactions, the faces of social partners transmit a variety of dynamic social information, including that related to health, arousal, emotional and attentional states, and communicative intentions (e.g., Hessels 2020; Jack and Schyns 2015). Monitoring such social information aids observers in quickly updating or inferring the current mental state of their social partners in free‐flowing interactions. Because verbal communication in infants is limited, face monitoring may be more important for inferring social partners' mental states in interactions with infants than in interactions with their adult counterparts. In fact, one previous study reported that maternal responsiveness to the change in the infant's direction of gaze and maternal responsiveness to the infant's object‐directed action was positively correlated with mothers' appropriate mind‐related comments (Meins et al. 2001). Considering that face‐looking is likely to be a prerequisite for maternal responsiveness, we expected that the mother's tendency to look at infants' faces would be associated with individual differences in mothers' appropriate mind‐related comments.

Moreover, the association between verbal measurement of mind‐mindedness and mothers' looking at their infants' faces may be grounded in more real‐time behavior‐level coordination within individuals. Previous studies have shown that gazing at social partners' faces is coordinated in a timely manner with speech, such as turn‐taking in adult conversations (Ho, Foulsham, and Kingstone 2015; Kendon 1967) or labeling directed at infants (Abney et al. 2020; Custode & Tamis‐LeMonda 2020). Considering that mind‐mindedness is operationalized as the proportion of mothers' speech that pertains to infants' mental states, caregivers may also align face‐looking with the timing of their mind‐related speech.

We investigated the real‐time relationship between mothers' gaze at their infants' faces and typical verbal mind‐mindedness in free‐flowing infant‐mother interactions. Mothers were asked to wear a head‐mounted eye tracker to measure their gaze during infant‐mother free‐play interactions. We calculated the percentage of time mothers looked at their infant's face when it appeared in the mother's field of view and performed appropriate mind‐related comments. We hypothesized that maternal looking at their infants' faces is suitable as a non‐verbal behavioral measure of mind‐mindedness and that the behavior constitutes a real‐time maternal mentalization process in infant‐mother interactions. If face‐looking contributes to maternal mentalization processes, mothers with higher verbal mind‐mindedness would look at their infants' faces more frequently than would mothers with less verbal mind‐mindedness. Furthermore, within individual mothers, face‐looking would tend to be aligned with mind‐related comments compared to other comments.

## 2 Methods

### 2.1 Participants

Forty full‐term infants (22 male, 18 female) and their mothers participated in the study. The average age of the infant participants was 11.82 months (standard deviation [SD] = 0.53; range = 11–13). We chose to study 11‐ to 13‐month‐old infants because previous studies have measured mind‐mindedness in infant‐mother interactions at this age (Laranjo et al. 2014) and have assessed mothers' micro‐level gaze behavior using a head‐mounted eye tracker (Suarez‐Rivera, Smith, and Yu 2019; Yu and Smith 2013, 2016 2017b). We performed sample size estimation using G*Power 3.1 (Faul et al. 2007), assuming an effect size of 0.432 for correlation analyses, giving a required sample size of 37 to achieve 80% power. An effect size of 0.432 was set according to the effect size reported in a previous study that investigated the relationship between mind‐mindedness and non‐verbal behavior measurement (Bigelow et al. 2015); 90% of the previously reported effect size was used to account for potential overestimation. Participants were recruited from among infants registered in our laboratory database, which was constructed by delivering flyers through a lab website. All participants were of Japanese ethnicity. Regarding mothers' educational attainment, 10% of the mother participants had completed graduate training, 60% had standard college or university degrees, 22.5% had completed at least 1 year of specialized training or partial college, and 7.5% had completed high school. Data from an additional five infant–mother dyads were excluded: three participants had eye‐tracking data that did not meet quality standards (low eye‐detection rate (i.e., data loss more than 30%), *N* = 2; failure to calibrate, *N* = 1), one infant became too fussy to complete the study, and one participant's data were lost owing to equipment failure. This study was conducted in accordance with the Declaration of Helsinki guidelines and was approved by the Behavioral Research Ethics Committee of the Osaka University School of Human Sciences (HB021‐032). The mothers of all participating infants provided written informed consent.

### 2.2 Data Collection

Data were collected in a laboratory playroom at Osaka University. The playroom contained seven toys and the entire room was filmed using two fixed cameras. The mothers were instructed to play with their infants as they would at home. They could play with any available toy. The play sessions lasted for 20 min.

Before the play session, mothers were asked to wear head‐mounted eye trackers (Tobii Glasses 3; Tobii Technology). The eye tracker scene camera captured the mother's first‐person field of view (95° horizontal × 63° vertical field of view), and two eye cameras per eye recorded eye movements at 50 Hz. The field of view was recorded at 1920 × 1080 pixels at 25 fps. Calibration was performed using eye‐tracking software (Tobii Glasses Controller). For calibration, mothers were instructed to gaze at a black‐and‐white target displayed on a calibration card for several seconds.

### 2.3 Data Processing

#### 2.3.1 Verbal Mind‐Mindedness

We quantified each mother's verbal mind‐mindedness following the mind‐mindedness coding manual Version 2.2 (Meins and Fernyhough 2015). First, we transcribed the mothers' speech recorded via the head‐mounted eye tracker verbatim into individual comments. Comments were defined as words, phrases, or sentences that could be distinguished based on semantic or temporal discontinuities. A silence duration of more than 1 s was defined as a temporal discontinuity. We used transcription software (Vrew, VoyagerX Inc.) to generate an approximate transcription. We manually checked the transcriptions and corrected errors.

Next, we categorized each comment as mind‐related or non‐mind‐related based on whether it referred to the infant's mental state (Meins and Fernyhough 2015). All comments that included an explicit internal state term referring to what the infants may be thinking, experiencing, or feeling were categorized as mind‐related comments. Comments about infants' perceptions (i.e., seeing, watching, looking, listening, touching) were classified as non‐mind‐related comments (e.g., “Look! It's red,” “What are you looking at?”). Each mind‐related comment was categorized as appropriate or non‐attuned. Appropriate mind‐related comments were those that (a) accurately captured the infant's current internal state (e.g., “You want the car.”—when the infant was reaching toward the car), (b) connected the infant's current internal state with similar events in the past or future (e.g., “Do you remember seeing an elephant at the zoo?”—when the infant was playing with a toy elephant), (c) suggested potential activities that the infant would like or want during interaction breaks (e.g., “Do you want to play with the ball?”), or (d) voiced what the infant would say if they could talk (e.g., “Thank you, I like this”—when the mother passed a toy to the infant). In contrast, non‐attuned mind‐related comments were those that (a) attributed an internal state that did not align with the infant's current behavior (e.g., “You are bored with that car”—when the infant was still actively playing with the car toy), (b) attributed internal states referring to a past or future event that was unrelated to the infant's current activity (e.g., ‘‘Do you want noodles for dinner?”—when no previous play or discussion focused on food), (c) suggested that the infant wants to start a new activity when the infant is already actively engaged in playing with something else, (d) in which the referent of the mother's internal state comment was not clear (e.g., ‘‘You like that’’—when the infant was not playing with or attending to any particular object or event).

A trained researcher who was blind to the hypotheses categorized the mothers' comments while viewing the recordings from fixed video cameras in conjunction with the transcriptions. A naïve coder who was also blind to the hypotheses categorized a randomly selected 25% of comments; intercoder agreement was 94.8% (*kappa* = 0.61). Finally, we scored the proportion of appropriate comments out of the total number of comments produced during the interaction as the verbal measurement of the mother's mind‐mindedness.

#### 2.3.2 Face‐Looking

We quantified maternal looking at infants' faces using video recordings from a head‐mounted eye tracker worn by mothers. First, we downsampled each video to a still frame every second (1 Hz). Subsequently, we asked two coders to identify images containing infants' faces. We operationalized the presence of a face as a face containing any three points from the left eye, right eye, nose, and mouth. A total of 48,014 downsampled images were coded and infants' faces were identified in 14,121 images. To assess intercoder reliability, 20% of the downsampled images were randomly selected and coded by the coders. Intercoder agreement was 95.2% (*kappa* = 0.90).

**FIGURE 1:**
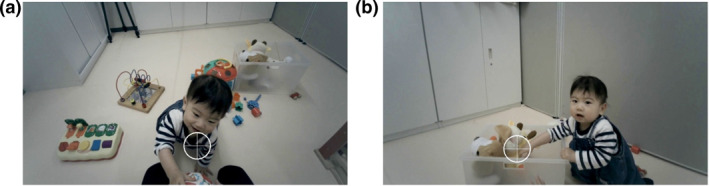
Images of a Mother Looking at Her Infant's Face. The white circle represents a gaze area of interest (AOI), defined as a circle of radius 4° centered on the point of gaze. The white cross‐hair represents the caregiver's point of gaze recorded by a head‐mounted eye‐tracker. The mother was defined to be looking at the infant's face when there was any overlap between the gaze AOI and the infant's face. (a) Face‐looking. (b) Not face‐looking.

Next, using images in which mothers' pupils were detected and that included their infants' faces, we checked whether mothers were looking at their infants' faces. Following the procedure used by Franchak, Kretch, and Adolph (2018), we drew a gaze area of interest (AOI) as a circular cursor of a radius of 4° and centered it on the mother's point of gaze. We asked two naïve coders to categorize the mothers' face‐looking state (face‐looking/not face‐looking) for each image based on any overlap between the face and gaze AOI (Figure 1). To assess intercoder reliability, 20% of the images were randomly selected and coded by the coders. Intercoder agreement was 99.6% (*kappa* = 0.99).

Finally, we calculated the proportion of images with face‐looking out of the images in which mothers' pupils were detected and that contained their infants' faces for each infant‐caregiver dyad. We scored the value as the face‐looking tendency of the mother.

### 2.4 Statistical Analysis

To examine the association between mothers' mind‐related comments and face‐looking, we performed two analyses using a general linear model (GLM) or general linear mixed model (GLMM) with a binomial error structure and a logit link function. The first analysis tested whether individual differences in face‐looking tendencies were associated with mothers' verbal mind‐mindedness, which addressed the study aim of determining whether looking at infants' faces could be one of the non‐verbal behaviors reflecting the mothers' mind‐mindedness. The second analysis tested whether the mothers' looking at their infants' faces was coordinated in a timely manner with mothers' appropriate comments about the infants' mental states. This analysis addressed the aim of determining whether a mother's face‐looking behavior constitutes a real‐time maternal mentalization process in infant‐mother interactions. Analyses were performed in R 4.4 (R Core Team 2024) using the lme4 package (Bates et al. 2014). This study's design and its analysis were not pre‐registered.

## 3 Results

### 3.1 Descriptive Statistics

Table 1 presents the descriptive statistics for the measures of mothers' comments and face‐looking. The mean number of comments was 343.30 (SD = 102.68), of which 5.77% (SD = 3.07) were appropriate and 0.41% (SD = 0.48) were non‐attuned. Mothers produced appropriate mind‐related comments more frequently than they did non‐attuned mind‐related comments (paired *t*‐test, t(39)=11.46,p<0.001).

**TABLE 1:** Descriptive statistics for the measures of mothers' comments and face‐looking.

Variable		Mean	SD	Min	Max
Comments	Total number of comments	343.30	102.68	143	542
	Appropriate mind‐related comments (%)	5.77	3.07	1.46	14.72
	Non‐attuned mind‐related comments (%)	0.41	0.48	0	1.55
Images	Mothers' pupils were detected (%)	92.15	5.86	75.42	99.92
	Containing infants' faces (%)	29.41	13.41	10.58	64.25
	Face‐looking in total images (%)	11.94	5.82	2.58	25.58
	Face‐looking in images containing infants' faces and mothers' pupils (%)	44.53	12.50	25.26	82.78

Regarding eye‐tracking measures, the mean proportion of images in which mothers' pupils were detected was 92.15% (SD = 5.86), and the mean proportion of images containing infants' faces was 29.41% (SD = 13.41). Mothers' pupils were detected in 92.30% of images containing infants' faces on average (SD = 5.62). After categorizing mothers' face‐looking states, the mean proportion of face‐looking out of the total images was 11.94% (SD = 5.82), and the mean proportion of face‐looking out of images containing infants' faces and mothers' pupils was 44.53% (SD = 12.50).

### 3.2 Relationship Between Mothers' Mind‐Related Comments and Face‐Looking Tendencies

**FIGURE 2:**
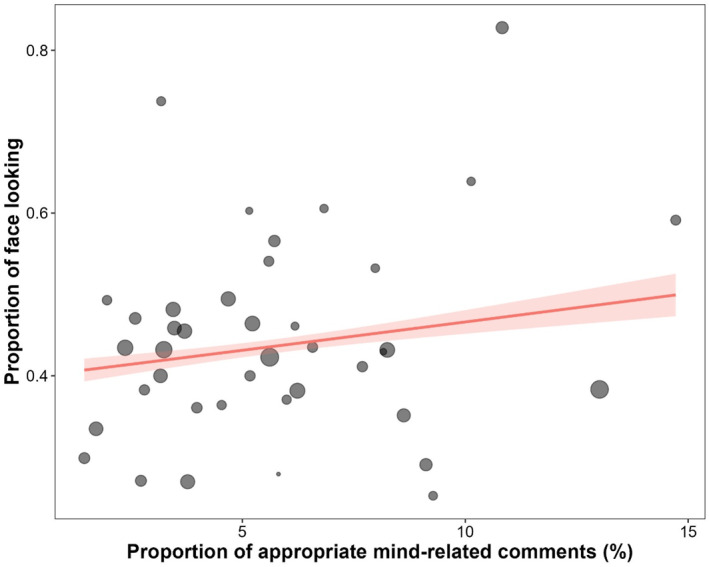
Relationship between the Proportion of Mothers' Looking at infants' Faces and the Proportion of Appropriate Mind‐Related Comments. The colored line shows the predicted values in the proportion of face‐looking from images in which face and gaze were detected. Symbols represent observed values obtained from individual mothers, with the size indicating the number of images containing infants' faces. Shaded regions represent 95% confidence intervals.

We performed GLM with a binomial error structure and a logit link function to examine the association between individual differences in face‐looking tendencies and mothers' verbal mind‐mindedness. The response variable was the proportion of maternal face‐looking in images containing infants' faces, and the explanatory variable was the proportion of appropriate mind‐related comments. The likelihood ratio test revealed a significant effect of appropriate mind‐related comments ($\chi^{2}\left(1\right)=25.47,p<0.001$). Mothers with a larger proportion of appropriate mind‐related comments were more likely to look at their infants' faces (Figure 2; Table S1). Mothers' face‐looking tendencies were positively associated with the proportion of appropriate mind‐related comments, which is a verbal measure of mothers' mind‐mindedness.

### 3.3 Real‐Time Coordination Between Appropriate Mind‐Related Comments and Face‐Looking

To examine whether mothers aligned their face‐looking with appropriate comments about infants' mental states, we coded the presence of any images with mothers' looking at infants' faces within a 5‐s time window, which spanned 2.5 s before and 2.5 s after the onset of each comment. All comments were categorized as appropriate mind‐related or other comments. We then compared the proportion of comments for which face‐looking occurred between appropriate mind‐related and other comments. If mothers' looking at infants' faces was coordinated with appropriate mind‐related comments, face‐looking was expected to be more frequently observed around appropriate comments than around other comments.

**FIGURE 3:**
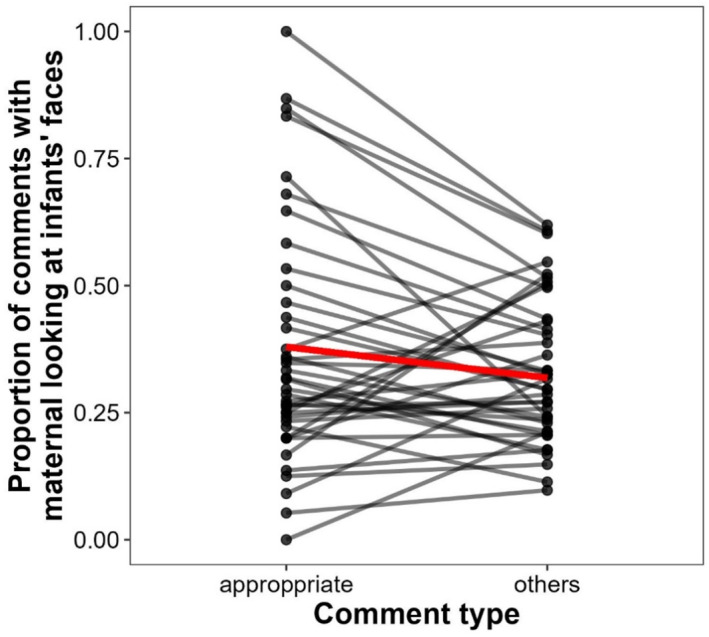
The Proportion of Comments Coinciding with Mothers' Looking at Infants' Faces by Comment Type. Symbols connected with a black line represent observed values from an individual mother. The thick red line represents predicted values of the proportion of comments coinciding with face‐looking.

We found that the proportion of comments coincident with face‐looking was significantly larger for appropriate comments than for other comments (Figure 3). We performed a GLMM with a binomial error structure and a logit link function to estimate the effect of comment type (appropriate/others) on the proportion of comments coincident with face‐looking. A likelihood ratio test revealed a significant main effect of comment type ($\chi^{2}\left(1\right)=10.92,p<0.001$; Table S2). To check the robustness of the finding, we tested the effects of comment type by changing the size of the time window around each comment from 4 to 6 s in steps of 1 s. We confirmed a significant effect of comment type in the same direction regardless of the time window size (ps<0.01; Figure S1). Taken together, caregivers were more likely to look at infants' faces coincident with appropriate comments than with other comments.

Moreover, we also tested whether the observed effect of comment type can be explained by random co‐occurrence between mothers' comments and face‐looking based on the occurrence rate of each behavior in a play session. We generated 1000 randomized datasets (RDs) by shuffling the stream of face‐looking and pairing it with the real stream of comments for each mother. The RDs preserved the occurrence rates of comments and face‐looking for each mother but lost the fine time structure between them. Thus, we used RDs as control datasets under the null hypothesis that comments and face‐looking occur independently in each play session. Each RD was fitted with the same statistical model as the original dataset, and we compared the observed effect size to the distribution of the effect size estimated from 1000 RDs. The observed effect of comment type was not in the range of 95% confidence intervals of the effect size estimated from RDs (Figure S2). Thus, mothers' tendency to coordinate face‐looking with appropriate comments over other comments was stronger than expected from random co‐occurrence between face‐looking and comments.

Finally, we also explored whether the proportion of appropriate comments coincident with face‐looking changed between the first and second halves of the 5‐s time window. However, we found no significant differences between before and after the comments ($\chi^{2}\left(1\right)=0.67,p=0.41$).

## 4 Discussion

We investigated whether maternal gaze at an infant's face is closely related to the verbal measurement of mind‐mindedness during infant‐mother free‐play interactions. Specifically, we examined the correlation between individuals and co‐occurrence within individuals between maternal gaze at the infant's face and typical verbal mind‐mindedness (i.e., appropriate mind‐related comments). We found that the caregivers who displayed more verbal mind‐mindedness looked at their infant's face more frequently. Moreover, their looking at infants' faces was coordinated in a timely manner with appropriate mind‐related comments compared with other comments. These results suggest that face‐looking is related to appropriate mind‐related comments at both inter‐ and intra‐individual levels.

We found that maternal face‐looking behavior was positively correlated with appropriate mind‐related comments. This result suggests that maternal looking at infants' faces is one of the non‐verbal behavioral manifestations of mind‐mindedness. Previous studies have demonstrated that individual differences in other types of caregivers' non‐verbal behaviors (e.g., mirroring behavior and PEM) are associated with individual differences in appropriate mind‐related comments (Bigelow et al. 2015; Shai and Meins 2018). Our finding is in line with previous studies suggesting that a mother's sensitivity to infants' mental states manifests in both non‐verbal behaviors and mind‐related comments. Moreover, our findings provide new evidence of how mothers coordinate non‐verbal behaviors with mind‐related comments in real‐time social interactions. In addition to showing the association between non‐verbal behavior and verbal mind‐mindedness as between‐individual correlations, which was also examined in previous studies (Bigelow et al. 2015; Shai and Meins 2018), the current study assessed this association as a within‐individual cooccurrence. Our findings show that maternal face‐looking is temporally and spatially related to appropriate mind‐related comments.

Interestingly, we found that face‐looking tended to align with mind‐related comments compared to other comments and that it could not be explained by random co‐occurrence between maternal face‐looking and comments. The findings suggest that maternal face‐looking may constitute a real‐time maternal mentalization process in infant‐mother interactions. Faces transmit information about what others are looking at (Frischen, Bayliss, and Tipper 2007) and how they feel or think (Baron‐Cohen, Wheelwright, and Jolliffe 1997). Therefore, looking at infants' faces might help decode these signals quickly and aid in adjusting to mothers' mind‐related comments accordingly.

What is the function of mothers' looking at infants' faces in alignment with their appropriate mind‐related comments? One possible function could be monitoring infants' states. Mothers may have tracked their infants' arousal, emotional, attentional states by looking at their faces to infer their mental states before producing mind‐related comments or to assess their response after the comments. Another possible function could be signaling communicative intentions to their infants. Mothers may have signaled their communicative intentions to their infants by looking at their faces to initiate mind‐related comments or maintain face‐to‐face communication even after the comments. These are known as the dual functions of social gaze and have been well documented in previous studies about social attention in face‐to‐face interactions (Cañigueral and Hamilton 2019; Hessels 2020). Given that social gaze supports the regulation of interaction, whether in a monitoring or signaling function, it is reasonable for mothers to look at the infant's face both before and after the mind‐related comments. Interestingly, we found no significant differences in the proportion of comments coincident with face‐looking before and after appropriate mind‐related comments. Thus, mothers' face‐looking may play an important role in the real‐time maternal mentalization process around the timing of mind‐related comments.

Our findings are also consistent with those of previous studies that emphasize the importance of mothers' face monitoring in language development. In object free‐play, mothers tend to produce an object label while looking at infants' faces and objects (i.e., triadic gaze; Abney et al. 2020; Custode & Tamis‐LeMonda 2020). Furthermore, individual differences in mothers' triadic gaze predict later infant vocabulary size (Abney et al. 2020; Tomasello and Farrar 1986). As a theoretical framework, Tamis‐LeMonda, Kuchirko, and Song (2014) proposed that mothers' labeling while monitoring their infants facilitates temporal, semantic, and pragmatic mapping between words and objects or actions of interest to infants. Similarly, mothers' mind‐related comments while monitoring their infants may facilitate temporal, semantic, and pragmatic connections between mental state words and the infant's corresponding situation or mental state. The accumulation of such experiences in infant‐mother interactions over developmental time may shape infants' feelings of security or understanding of others' mental states, possibly laying a foundation for the later development of attachment (Meins et al. 2001, 2012) or theory of mind (Meins et al. 2002, 2003).

Although face‐looking was coordinated with mothers' comments about infants' mental states, little is known about the regions within infants' faces at which mothers looked. Previous studies have shown that the allocation of gaze to facial features depends on the observers' ongoing tasks, including face identity recognition (e.g., Ueno et al. 2021), speech perception (e.g., Buchan, Paré, and Munhall 2007), and emotion recognition (e.g., Vaidya, Jin, and Fellows 2014). When focusing on inferring the mental states of infants, which facial features are relevant to the maternal mentalization process? One possibility is that the eyes are particularly relevant. Information from the eyes has been considered vital, especially for recognizing mental states rather than basic emotions (Baron‐Cohen, Wheelwright, and Jolliffe 1997; see also Back, Ropar, and Mitchell 2007). Consistent with this perspective, meta‐analyses of functional brain imaging studies found that the brain activation area for attributing mental states from the eyes is part of the core brain network for theory of mind or mentalization (Mar 2011; Schurz et al. 2014). Thus, the eyes may be an important facial feature during the maternal mentalization process. Further studies using automatically generated facial AOIs (Hessels et al. 2018) are required to reveal which facial features are processed during real‐time maternal mentalization.

One contribution of the current study is to demonstrate the utility of measuring maternal face‐looking as one of the non‐verbal behavioral manifestations of a mother's mind‐mindedness in a real‐time social interaction. Maternal face‐looking, measured using a head‐mounted eye tracker, can be assessed reliably and allows researchers to examine the real‐time relation to various ongoing behaviors of infants or mothers in free‐flowing interactions. Moreover, the maternal face‐looking measure is possibly practical in terms of the cost of coding; although the current study used manual coding for the assessment of face‐looking, the use of an automatic face detection algorithm (e.g., Hessels et al. 2018) would accelerate the process. In the future, this advantage would help researchers understand how caregivers organize behaviors, including face‐looking, to infer the current mental states of infants in their everyday environments, using a day‐long recording of mothers' sensory experiences. This method has the potential to be developed into clinical applications, such as interventions to help caregivers at risk for caregiving difficulties look at their infants' faces to facilitate their mentalization processes.

A limitation of this study is that we did not test whether looking at faces predicts future development in infants, as previous studies have shown with verbal mind‐mindedness. Previous studies have shown that appropriate mind‐related comments are the most sensitive among several measurements, including non‐verbal behaviors (e.g., mothers' response to changes in infants' line of gaze), to predict later development, such as secure attachment and theory of mind (Meins et al. 2001). In this study, face‐looking was closely related to simultaneously measured appropriate mind‐related comments, but the extent to which face‐looking predicts later infant development is unknown. To understand the usefulness of face‐looking as a nonverbal measure of mind‐mindedness, it is important to compare it with verbal mind‐mindedness in terms of predicting later development. However, the strengths of the current study's methodology, which permits measurement of face‐looking in more ecological contexts, may contribute to this issue in the future. In recent years, there have been efforts to evaluate maternal behaviors in the home environment as opportunities for infant development, including eye contact, object play, and infant‐directed speech (Custode et al. 2020; Roy et al. 2015; Suarez‐Rivera et al. 2022; Yamamoto, Sato, and Itakura 2019, 2020). Further studies should elucidate how experiences of mothers' face‐looking in everyday life guide the later development of their infants, including their attachment and theory of mind.

Another limitation of the current study is that we did not focus on mothers' looking behavior toward regions other than faces, such as toward infants' manual actions (Figure 1b). As gaze toward manual actions on objects is associated with understanding action goals or intentions (e.g., Flanagan and Johannson 2003; Kanakogi and Itakura 2011), in addition to looking at infants' faces, looking at infants' manual actions may also contribute to maternal mentalization processes. Indeed, previous studies have demonstrated that mothers' attachment or theory of mind is predicted by their responses to an infant's object‐directed actions (Meins et al. 2001, 2003). In infant‐mother interactions, infants' manual actions guide eye movements and joint attention (Yu and Smith 2013, 2017a, 2017b), and lead to mothers' naming utterances (Custode & Tamis‐LeMonda 2020; Tamis‐LeMonda, Kuchirko, and Tafuro 2013; West and Iverson 2017). Considering that mothers do not always have visual access to their infant's frontal face, the presence of redundant, multiple social information such as infants' faces and manual actions, may allow robust and flexible real‐time maternal mentalization, even in open‐ended free‐flowing infant‐mother interactions.

The current study also had some procedural limitations. Although significant associations between mothers' looking at infants' faces and verbal mind‐mindedness were found, it is possible that the sampling design was underpowered. The effect size used for sampling size estimation in the current study was higher than that estimated in a meta‐analysis on maternal mentalization and sensitivity (Zeegers et al. 2017). A more robust sample size design will be required to investigate relationships between non‐verbal behaviors and mothers' mind‐mindedness in the future. Moreover, compared to the high percentage of agreement for categorization of mothers' comments, the kappa coefficient was modest (*kappa* = 0.61 with 94.1% agreement). This may be owing to uneven distributions of codes across the categories of mothers' comments. Although the reliability value in the current study was acceptable (McHugh 2012) and the proportion of each category of mothers' comments was comparable to those in previous studies using similar coding schemes (e.g., Meins et al. 2012; Shai and Meins 2018), it is important to note that our results rely upon the categorization of mothers' comments, which was not in perfect agreement. Further research is needed to understand the role of maternal face‐looking in real‐time infant‐caregiver interactions, including examining the relationship of maternal face‐looking not only with mothers' comments but also with other non‐verbal measures related to mind‐mindedness.

Notwithstanding these limitations, to the best of our knowledge, this is the first study to examine the real‐time relationship between mothers' looking at infants' faces and verbal mind‐mindedness in free‐flowing infant‐mother interactions. Mothers were likely to look at infants' faces when they appropriately commented regarding the infant's mental state. Consistent with this timely coordination between face‐looking and appropriate mind‐related comments, mothers who made more appropriate mind‐related comments were more likely to look at infants' faces. In open‐ended free‐flowing infant‐mother interactions, infants' attentional and emotional states change from moment to moment. To be sensitive to and respond to what infants are interested in, or what they feel or think about, mothers need to access or update the infant's state through visual exploration. The current study suggests that looking at infants' faces supports mothers' comments about infants' mental states and sheds new light on real‐time behaviors underlying mothers' mentalization processes.

## Author Contributions

**Hiroki Yamamoto:** conceptualization, methodology, software, formal analysis, investigation, visualization, data curation, writing–original draft, writing–review and editing. **Nagomi Sunahara:** investigation, writing–review and editing. **Yasuhiro Kanakogi:** conceptualization, resources, supervision, writing–original draft, writing–review and editing, project administration, funding acquisition.

## Conflicts of Interest

The authors declare no conflicts of interest.

## Supporting information

Supporting Information S1

## Data Availability

The datasets, codes, and translated coding manual for the mothers' comments used in this study are available from the GitHub repository (https://github.com/dororo1225/caregivers‐mm).
