# Supplementary material for: Face‐Looking as a Real‐Time Process in Mind‐Mindedness: Timely Coordination Between Mothers' Gaze on Infants' Faces and Mind‐Related Comments
Source: Infancy. 2024 Dec 1;30(1):e12644. doi: 10.1111/infa.12644 (PMC11608580; doi:10.1111/infa.12644)
Supplement: Supplementary file 1 — Supporting Information S1 [file INFA-30-0-s001.docx]

| Effect | Variable | Estimate | SE | 95% CI | p |
| --- | --- | --- | --- | --- | --- |
| Fixed | **Intercept** | **-0.26** | **0.02** | **[-0.29, -0.22]** | **< 0.001** |
|  | **Proportion of appropriate mind-related comments** | **0.09** | **0.02** | **[0.06, 0.12]** | **<0.001** |

**Supplementary Table S1**

*Parameter Estimates for a Model Predicting Mothers’ Face-Looking from the Proportion of Appropriate Mind-Related Comments*

*Note*. Significant fixed effects are indicated in bold. The explanatory variables are scaled and centered on the mean.

**Supplementary Table S2**

*Parameter Estimates for a Model Predicting the Proportion of Comments Coincident with Mothers’ Face-Looking by Comment Type*

| Effect | Variable | Estimate | SE | 95%CI | p |
| --- | --- | --- | --- | --- | --- |
| Fixed | **Intercept** | **−0.49** | **0.13** | **[−0.75, −0.23]** | **< 0.001** |
|  | **Comment type [others]^a^** | **−0.27** | **0.08** | **[−0.43, −0.11]** | **< 0.001** |
| Random | Intercept (Mother) | 0.67 | 0.08 | [0.53, 0.84] | - |

*Note*. Significant fixed effects are indicated in bold. For random effects, the standard deviation of the parameters according to each random-effect grouping is shown.

^a^ Comment type was dummy coded with the reference category “appropriate.”


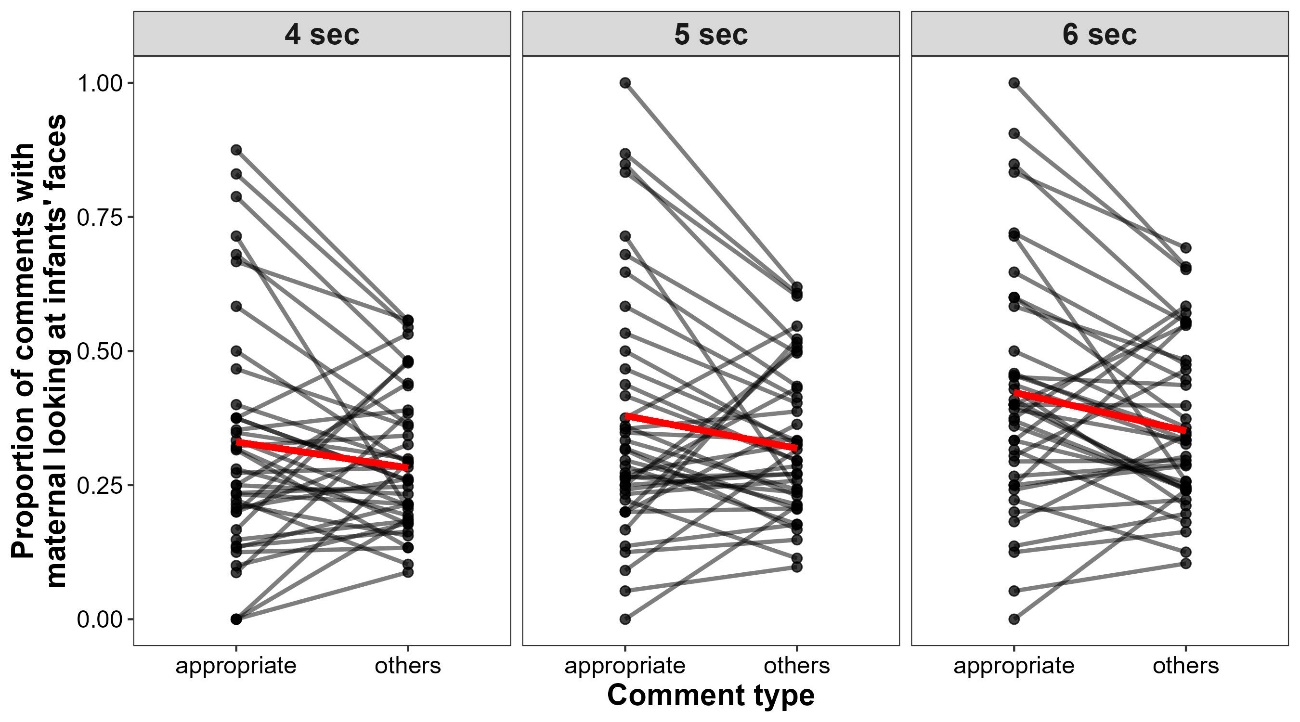


**Supplementary Figure S1**

*The Proportion of Comments Coincident with Maternal Looking at Infants’ Faces by Comment Type for Each Time Window.*

*Symbols connected with a black line represent observed values from an individual mother. The thick red line represents predicted values of the proportion of comments coincident with face-looking. Each subplot represents the observed and predicted values obtained when the size of the time window around a comment is changed from 4 s to 6 s in 1 s increments.*


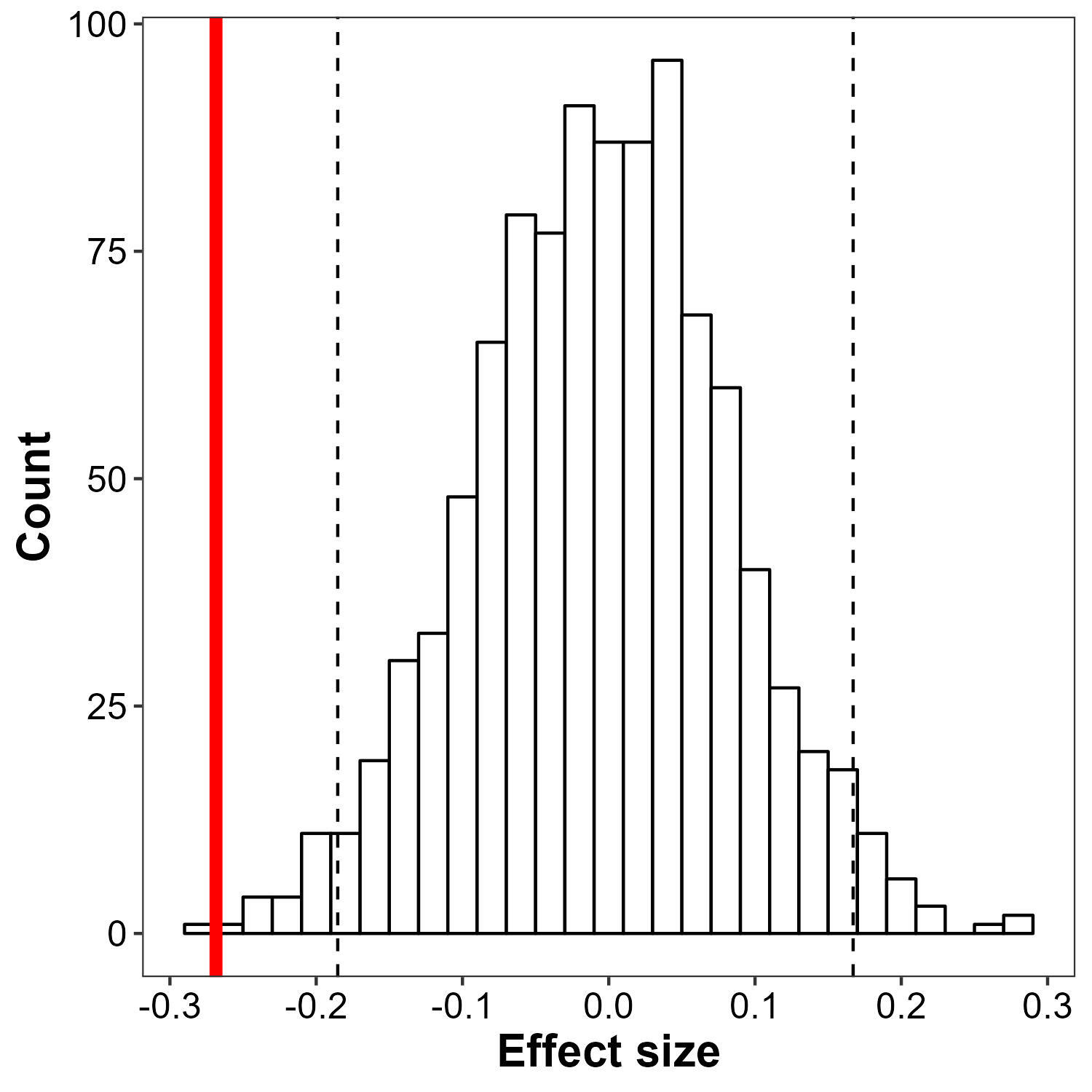
**Supplementary Figure S2**

*The Observed Effect Size of Comment Type and the Distribution of the Effect Size Estimated from 1,000 Randomized Datasets.*

*The histogram represents the distribution of effect size estimated from 1,000 randomized datasets. The dotted vertical lines represent the lower and upper boundaries of the 95% confidential interval. The thick red vertical line represents the observed effect size of comment type. Note that effects of comment type were dummy coded with the reference category “appropriate.”*
